# Protein turnover in plants—cost of synthesis, repair, and crop engineering opportunities

**DOI:** 10.1093/plphys/kiag481

**Published:** 2026-07-08

**Authors:** Anastasia J I Högerl, Sudarshan Gnanamani, Ulschan Bathe

**Affiliations:** Biotechnology of Horticultural Crops, TUM School of Life Sciences, Technical University of Munich, Liesel-Beckmann-Straße 1, Freising 85354, Germany; Biotechnology of Horticultural Crops, TUM School of Life Sciences, Technical University of Munich, Liesel-Beckmann-Straße 1, Freising 85354, Germany; Biotechnology of Horticultural Crops, TUM School of Life Sciences, Technical University of Munich, Liesel-Beckmann-Straße 1, Freising 85354, Germany

## Abstract

Protein lifespan is shaped by plant developmental stage and cellular requirements; both are fairly well understood processes. Beyond regulated degradation, chemical damage is another major cause of protein turnover, yet it is an understudied area in plant biology and crop engineering. The associated respiratory costs limit harvestable crop yield because the degradation and resynthesis of inactivated proteins consume carbon that then cannot be used for biomass production. Approximately half of the carbon fixed by plants is lost through respiration; of that, protein turnover consists of ∼25% to ∼40%. In this review, we examine the importance of spontaneous protein modifications arising under both optimal and environmental stress conditions; the latter can elevate protein turnover costs, negatively impacting crop yield. While homeostasis of most proteins following damage is likely maintained via de novo synthesis, some molecular mechanisms exist in cells to prevent and repair protein damage. They offer a substantial energetic advantage over resynthesis. We illustrate these benefits with specific examples and quantify their associated costs. Finally, understanding the causes, energetic consequences, and repair mechanisms of protein damage can inform strategies to improve crop performance. We suggest potential protein targets and synthetic biology approaches to be exploited for future crop engineering.

## Introduction

**Figure 1:**
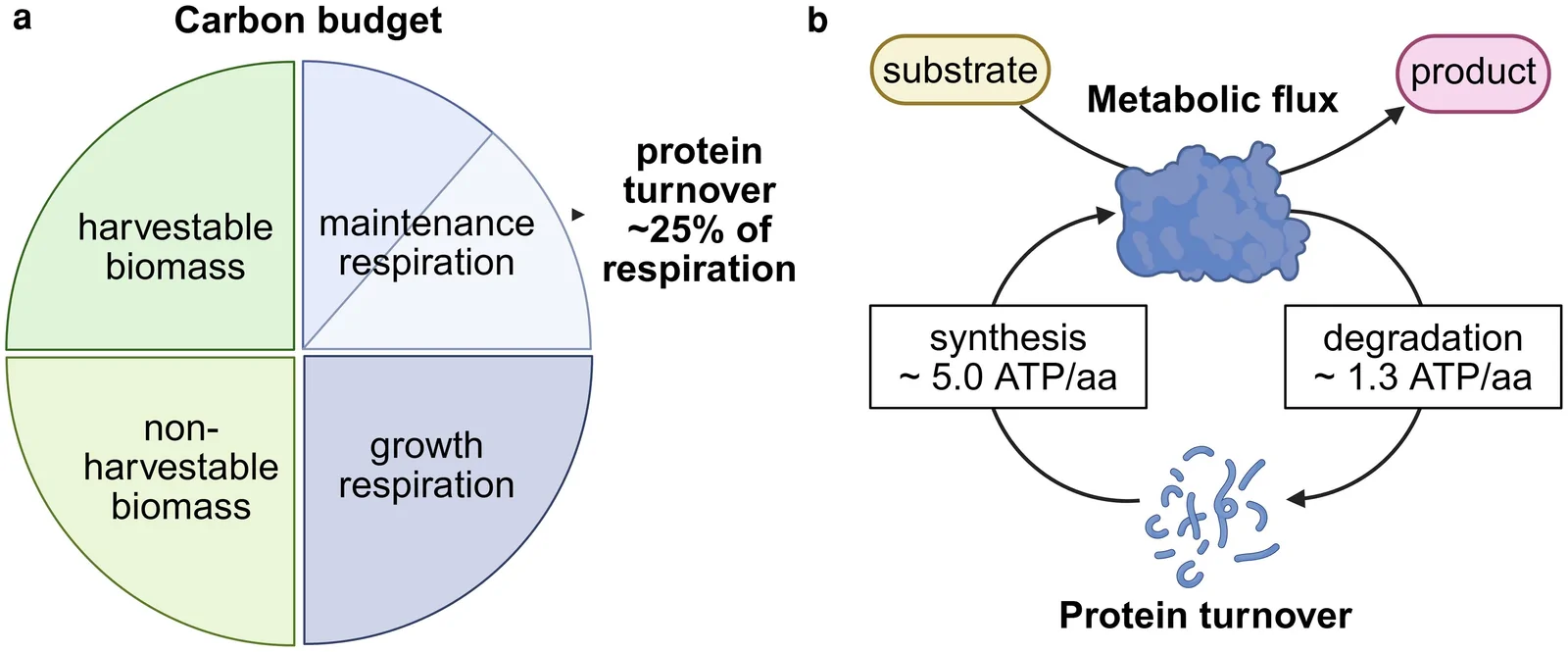
Illustration of the plant carbon budget and costs of protein turnover. a) C fixed by photosynthesis is equally used for biomass production (left part of pie chart) and respiration (right part of pie chart). In this example, protein turnover consumes 25% of respiration. b) Protein degradation and resynthesis cost ∼6.3 ATPs per amino acid. The figure was created in BioRender (https://BioRender.com/5awtzf9).

Carbon (C) fixed by photosynthesis is spent on biomass production and respiration; both account for about half of the C/energy budget of a plant (Fig. 1a) (Bathe et al. 2023). While C for biomass production is fixed in tissues, respiration returns C as CO_2_ (efflux) to the atmosphere, which means that half of the photosynthetically produced carbohydrates are lost instead of being stored as biomass. Respiration is essential though energy expensive, providing ATP and NAD(P)H for diverse cellular processes, including growth respiration (eg nutrient assimilation, synthesis of new structural biomass) and maintenance respiration (eg maintenance of gradients, cell walls, and nucleic acids) (Kozlowski and Pallardy 1997). One of the most ATP-draining respiratory processes is protein turnover, which is the continuous degradation and resynthesis of proteins—a dynamic process that is needed to maintain cell function and homeostasis. The costs can range from 10% to 75% of total respiration (de Visser et al. 1992). During turnover, proteins are degraded and cut into their amino acid parts, some of which are then recycled for de novo synthesis. The costs per amino acid are ∼1.3 ATPs for degradation and ∼5 ATPs for protein assembly (Fig. 1b) (Amthor 2000). For proteins that are synthesized in the cytosol and translocated to organelles, additional costs of ∼1 to ∼5 ATPs per amino acid are required for transport across membranes, increasing total costs per protein (Møller et al. 2007; Shi and Theg 2013). For ease of calculation (eg not all cytosolic synthesized proteins are transported to organelles, and some proteins are produced in organelles), we use 6.3 ATPs per amino acid to calculate minimum turnover costs. For example, replacing a single midsize protein of 500 amino acids requires at least 3,150 ATPs. These considerations assume that amino acids are used for the assembly. It should be noted that more costs can arise from nutrient assimilation, amino acid synthesis, or active transport.

Advances in proteomics and stable isotope labelling have allowed the quantitative study of protein turnover rates in plants and other organisms, even down to the single cell level (Sabatier et al. 2025; Li et al. 2026). For example, plants turn over most of their proteins within hours or days of their synthesis with a median turnover number of 1 to 2 days depending on growth conditions (Li et al. 2017; Li et al. 2022). Related energy requirements are estimated to be in the range of 10% to 40% of a plant's total C budget (de Visser et al. 1992; Scheurwater et al. 2000; Noguchi et al. 2001; Li et al. 2017), and Fig. 1a illustrates a simplified case of 12.5% (= 25% of respiration).

Cells degrade proteins because of developmental regulation and signaling, to mobilize resources, to adapt to environmental changes, for quality control and because of damage (Box 1). When proteins are damaged or misfolded, they must be replaced, which involves identifying and degrading them to prevent their accumulation. The more often there is damage, the more costs arise to degrade and replace essential proteins (Box 2).

Box 1 **Protein modifications that lead to damage**

**Figure 2:**
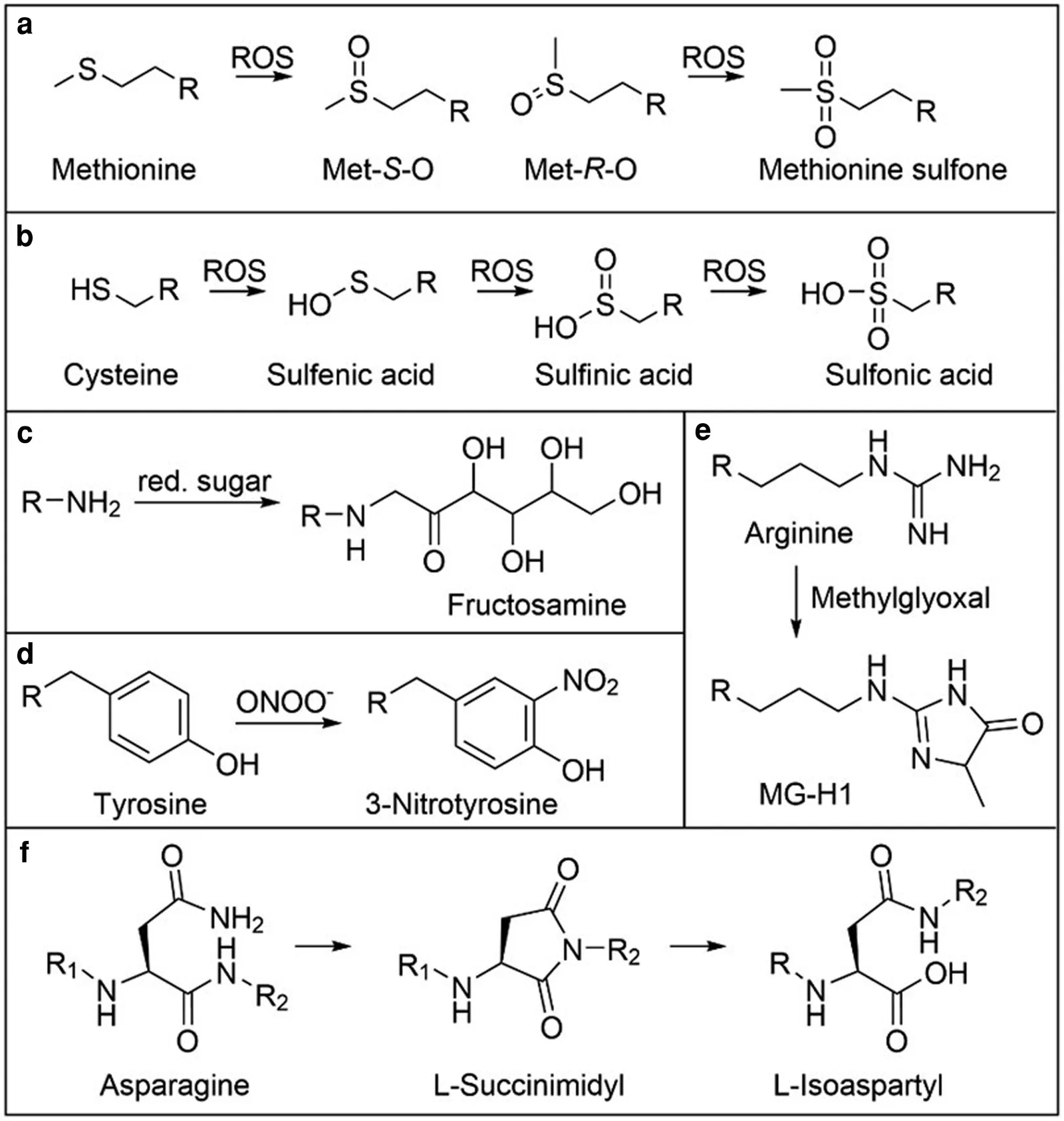
Examples of protein modifications that cause damage. a) Methionine oxidation. b) Cysteine oxidation. c) Glycation. d) Tyrosine nitration by peroxynitrite (ONOO^−^). e) Arginine carbonylation. f) Spontaneous deamination of asparagine. MG-H1: MG-derived hydroimidazolone 1.

Diverse protein modifications (Fig. 2) contribute to the chemical and functional complexity of proteomes (Keenan et al. 2021). They impact protein activity, localization, and stability through changes in hydrophobicity, charge, reactivity, structure, and folding. Some modifications can damage proteins, and their accumulation over time (protein fatigue) triggers protein degradation (see Outstanding questions) (Møller et al. 2007; Cuanalo-Contreras et al. 2022).

Spontaneous protein damage is often driven by reactive molecules which are byproducts of aerobic metabolism. Reactive oxygen species (ROS; eg hydroxyl radicals, superoxide anions, singlet oxygen, hydrogen peroxide) and reactive nitrogen species (RNS; eg nitric oxide, peroxynitrite) can either directly damage amino acid residues, primarily on the protein surface, or indirectly by oxidatively modifying carbohydrates or lipids that subsequently react with proteins (Møller et al. 2007; Fangue-Yapseu et al. 2022; Yao et al. 2026). Surface-exposed sulfur-containing amino acids, ie methionine and cysteine, function as ROS scavengers, thereby protecting other critical amino acid residues, and they may serve as molecular switches (Hou et al. 2024; Loiseau et al. 2025). Methionine oxidation (Fig. 2a) forms reversible methionine sulfoxide (Met-*R*-O and Met-*S*-O) or irreversible methionine sulfone, while cysteine oxidation produces thiyl radicals, unstable sulfenic acids, sulfinic acid, and sulfonic acid (Fig. 2b), reducing protein stability (Nauser et al. 2004; Chang et al. 2010; Iglesias-Baena et al. 2011; Jacques et al. 2015). Amino acids buried within proteins can be targets of oxidation too; for example, oxygen can be trapped within protein cavities during folding and is converted into ROS through blue-light irradiation of tryptophan, which can damage proximal amino acid residues (Kim et al. 2024).

Other protein modifications include glycation yielding fructosamines (Fig. 2c) (Lokhandwala et al. 2024), nitration affecting protein activity and cell signaling (Fig. 2d) (Yin et al. 2024), carbonylation that irreversibly introduces aldehydes or ketones (Fig. 2e) (Møller et al. 2011; Rao and Møller 2011; Fangue-Yapseu et al. 2022), as well as deamination and dehydration of asparagine and aspartate, respectively, followed by hydrolyzation to L-isoaspartyl (Fig. 2f) (Ogé et al. 2008).

Proteins also incur self-inflicted damage from their catalytic activity or reactive substrates, which is a major but understudied mode of enzyme failure concerning about half of central metabolic enzymes (Hanson et al. 2021). It is the random and stochastic damage to an enzyme's active site from a catalytic misfire or attack by a substrate or product (eg photoreactive, carbonyl and dicarbonyl, thiol, unstable, and radical metabolites) resulting in self-inactivation. The thiazole synthase is an extreme example, which catalyzes only a single reaction before being inactivated, ie it performs a suicide mechanism by donating the sulfur atom of an active-site cysteine to the product (Joshi et al. 2020).

Box 2 **Energy requirements of protein degradation in plants**

Protein degradation is crucial to prevent accumulation of protein aggregates and to preserve cell integrity. Energy-dependent steps depend on vesicle size, transport distance, and cargo. Plants use proteasomal and vacuolar routes to degrade cytosolic proteins. For detailed mechanisms, we direct the reader to reviews by others (Kraus et al. 2024; Petersen et al. 2024; Arkinson et al. 2025). The proteasomal pathway is tightly regulated and specific to proteins. It uses ubiquitin, a highly conserved 76-amino-acid polypeptide, to mark proteins for degradation (Tang et al. 2024). Ubiquitination involves E1 activating enzymes, which require one ATP per ubiquitin molecule to activate it for transfer (Lee and Schindelin 2008). E2 conjugating enzymes and E3 ligases then attach the activated ubiquitin to its substrate in an energy-independent manner. The protein target can be polyubiquitinated; thus, the process consumes multiple ATPs, before the 26S proteasome degrades it (Wang et al. 2022c). In the proteolytic complex made of a 20S core particle and 19S regulatory caps, ATP is hydrolyzed to recognize polyubiquitinated proteins, which are subsequently unfolded and translocated into the 20S core for digestion (Kandolf et al. 2022).

The vacuolar route degrades endocytosed extracellular material, plasma membrane proteins, large protein aggregates, organelles, and nucleic acids. It involves endocytosis and autophagy, which generate clathrin-coated vesicles and autophagosomes that are transported via endosomal intermediates or directly to the vacuole. Vesicle formation and autophagosome biogenesis require ATP for membrane remodeling, lipid phosphorylation, and protein complex assembly. For example, clathrin uncoating requires ATP hydrolysis by chaperons (Chatukuta et al. 2018), and autophagosome formation involves ATP-dependent kinase activities (eg ATG1/ULK complexes) and ubiquitin-like conjugation systems (ATG8/ATG12) (Huang et al. 2019; Aniento et al. 2022). While no fixed cost per vesicle can be defined, these steps can be considered to consume tens to hundreds of ATP molecules per vesicle or autophagosome, depending on size and complexity. The intracellular trafficking along the cytoskeleton requires additional energy, namely one ATP per 8-nm transport distance (Coy et al. 1999). Once endosomes or autophagosomes arrive at the vacuole, multiple ATPs and GTPs are needed per fusion event to release the cargo into the vacuolar lumen (Takemoto et al. 2018).

In plant mitochondria and chloroplasts, protein degradation is autonomous from the cytosolic pathways and involves ATP-dependent proteases, ATP-independent proteases, and oligopeptidases (Huang et al. 2020); some of them are likely specific to carbonylated substrates (Janska et al. 2010; van Wijk 2015; Smakowska et al. 2016). Individual costs vary between 0.1 and 2 ATPs per amino acid for substrate unfolding and translocation (Baker and Sauer 2006). Because mitochondria harbor some of the fastest turning-over proteins (eg HSP70-1 and FTSH3) (Nelson et al. 2013), turnover in this compartment is estimated to consume up to 10% of a plant's total energy budget (Møller et al. 2007).

The lifetime of a catalytic enzyme and how many reactions it can perform till its replacement are important features that contribute to its metabolic costs. For example, abundant but damage-prone, thus short-lived, enzymes are costly to plants because they must be replaced at a higher rate. They consume energy that cannot be used to build biomass, eg negative correlations were found between Arabidopsis growth and protein turnover (Ishihara et al. 2017). In vitro, the unitless metric “total turnover number” (TTN) quantifies the number of product molecules formed in the enzyme's working lifetime. It is often used in industry to evaluate an enzyme's catalytic efficiency in relation to its stability under a given condition (Bommarius 2023). In vivo, the “catalytic cycle till replacement” (CCR) is more suitable as it refers to an enzyme's functional lifetime in a biological system in which reaction partners, cellular regulation, metabolic pathways, and networks are more complex than in in vitro settings (Hanson et al. 2021). Using estimates for *Arabidopsis thaliana*, *Saccharomyces cerevisiae*, and *Lactococcus lactis*, Andrew Hanson and coworkers provided insights indicating that CCR can be correlated with the chemistry performed. For example, enzymes with low CCR values often have reactive substrates, products, and cofactors that can lead to protein damage, eg by “mechanism-based self-inactivation” (Bathe et al. 2021). The available CCR data was created under regular growth conditions, eg no biotic or abiotic stress was applied. It is likely that CCR values under both abiotic and biotic stresses decrease as such conditions favor protein damage and therefore turnover (see Outstanding questions) (Abukhalaf et al. 2023; Fan et al. 2024).

To prevent protein damage and to reduce unnecessary turnover, nature evolved mechanisms to scavenge reactive metabolites. They help to protect cellular structures, including proteins, from damage. Some of the enzymes involved display remarkable catalytic efficiency (Parmar et al. 2026), indicating strong evolutionary selection pressure on such pathways. Additionally, cells developed a limited number of repair mechanisms to cure damaged proteins. It is likely that most damaged proteins undergo recycling on the single amino acid level rather than on the polypeptide level. In this review, we discuss what causes protein damage, how protein damage can be prevented or repaired, what the related costs are, and what the engineering potential is to boost crop yield.

## Cellular mechanisms to prevent protein damage and its costs

Because many metabolites in plant cells can be harmful—with reactive oxygen species (ROS) being at the forefront—nature developed mechanisms to deal with some of them. We direct the reader to comprehensive reviews about metabolite damage and control mechanisms in plants (Møller et al. 2007; Hanson et al. 2016) and highlight here only antioxidant protection as well as cost considerations.

### Antioxidant metabolites

Plant cells are continuously exposed to ROS, for example, during light stress and pathogen defense reactions (Kuběnová et al. 2023; Zhao et al. 2024). Antioxidant metabolites help plants to cope with the oxidative stress by protecting cellular structures. Most plants synthesize protective compounds such as ascorbic acid, glutathione, tocopherols, flavonoids, proline, and carotenoids (Zhao et al. 2022b; Pirzada et al. 2024; Vives-Peris et al. 2024; Kreszies et al. 2025). They are classified as primary antioxidant metabolites because they derive from central metabolism and are produced constitutively. In contrast, specialized plant metabolites are often stress induced, and the biosynthesis includes specialized pathways that evolved in a limited number of plant species or families. For example, derivatives of the diterpenoid carnosic acid are specific to *Lamiaceae*, and antioxidant flavonoids such as vitexin and luteolin were identified in citrus species (Rao et al. 2023; Frey et al. 2025).

The production of antioxidant metabolites consumes resources, but their role in safeguarding cellular structures provides a net benefit to the plant, particularly when antioxidant metabolites can be recovered after scavenging ROS. For example, 2 molecules of glutathione are needed to detoxify one molecule of hydrogen peroxide. The biosynthesis from glutamate, cysteine, and glycine requires 2 ATPs per molecule of glutathione (with additional costs to make the 3 substrates), thus totals 4 ATPs as a minimum (Lu 2013). In contrast, investment in regeneration of the oxidized form glutathione disulfide consumes only one NAD(P)H (equivalent to ∼1.5 to ∼2.5 ATPs [Rasmusson et al. 2004; Hinkle 2005]) to recover 2 glutathione molecules (Vanoni et al. 1990). This equals ∼0.75 to ∼1.25 ATPs per glutathione molecule. ROS has certainly been one of the most prominent causes of (protein) damage in cells; therefore, it comes as no surprise that plants evolved mechanisms to regenerate antioxidants to benefit from reduced costs.

### Antioxidant enzymes

In addition to antioxidant metabolites, plants express a wide range of antioxidant enzymes to scavenge various ROS. They include superoxide dismutase (SOD), catalase (CAT), peroxidases, glutathione peroxidase, glutathione reductase, ascorbate peroxidase, thioredoxin (Trx), and peroxiredoxin (Mittler et al. 2004). For example, SOD converts superoxide radicals to hydrogen peroxide and molecular oxygen. Subsequently, 2 molecules of hydrogen peroxide can be converted into 2 molecules of water and one molecule of oxygen by CAT and peroxidases. Antioxidant enzymes have highly reactive substrates, and some of the resulting oxidations on the enzymes' methionine residues can be reversed by methionine sulfoxide reductase (MSR; see Protein repair mechanisms and the cost benefit over resynthesis). Although most hyperoxidized antioxidant enzymes must be replaced by degradation and resynthesis, the sulfinic acid derivative of peroxiredoxin represents an exception because it can be repaired through sulfiredoxin (Srx) (Iglesias-Baena et al. 2011). This reaction allows for a cost benefit over resynthesis of more than 1,200-fold (Table 1).

**Table 1:** Examples of plant protein repair enzymes and related direct or indirect costs.

Name	Mechanism	Cost	ATP equivalent	Cost of resynthesis relative to repair	Refs.
MSR^a^	Reductase, reduction of free or peptide-bound Met-O	1 NADPH per Met-O	2.5 ATPs	>1,000×	Tarrago et al. (2009)
Trx system	Reductase, NADPH-driven reduction of Trx	1 NADPH per Trx	2.5 ATPs	∼460×	Laloi et al. (2001)
Srx system	Reductase, ATP-dependent reduction of hyperoxidized peroxiredoxin	1 ATP	2 ATPs	∼1,260×	Iglesias-Baena et al. (2011)
Grx system	Reductase, NADPH-driven reduction of GSSG	1 NADPH per GSSG	2.5 ATPs	∼25×	Berkholz et al. (2008)
PIMT^a^	Methyltransferase, methyl ester formation to initiate aspartate regeneration	1 AdoMet per 0.3 isoaspartyl residues	∼22 ATPs per fully repaired L-isoaspartyl residue^b^	>100×	Skinner et al. (2000); Takusagawa et al. (1996); Roje et al. (1999)
FN3K	Kinase, phosphorylation of fructosamines to deglycate proteins	1 ATP per fructosamine	1 ATP	>1,000×	Shrestha et al. (2020)
Protein disulfide isomerase^a^	Isomerase, thiol-disulfide exchange via active-site cysteine pairs	1 FAD per disulfide bond	1.5 ATPs	>1,000×	Darby et al. (1994)
Peptidyl-prolyl *cis–trans* isomerases	Isomerase, *cis–trans* isomerization of prolyl bonds	None	—	Optimal^c^	Ingelsson et al. (2009)
sHsp–Hsp70 system	sHsp: Chaperon; followed by ATP-driven refolding of proteins: Hsp70	5 ATP per substrate	5 ATPs	∼700×	Sharma et al. (2010); Shi et al. (2025)
Hsp60	Chaperonin, ATP-driven refolding of proteins	7 ATP per cycle	7 ATPs	20 to 50×	Martin et al. (1991)
Hsp100/ClpB	Chaperon, ATP-driven protein quality control	Not well understood	6 to multiple hundreds ATPs	Dependent on substrate	Doyle and Wickner (2009); Hoskins et al. (2009)

The repair mechanisms of the listed enzymes allow reduced cost relative to their substrate's de novo synthesis. Some of them do not have direct repair costs, but the regeneration or resynthesis of cofactors requires energy. One NADPH/NADH can yield ∼1.5 to ∼2.5 ATPs (Rasmusson et al. 2004; Hinkle 2005), and 2.5 ATPs are used here for ease of comparison to calculate the minimum cost of resynthesis relative to repair. Met-O: methionine sulfoxide; Grx: glutaredoxin; GSSG: glutathione disulfide; PIMT: protein-L-isoaspartyl methyltransferase; (s)Hsp: (small) heat shock protein.

^a^The reaction requires no direct ATP, NADPH, or FAD supply, but regeneration of the repair enzyme or cofactors does.

**Figure 3:**
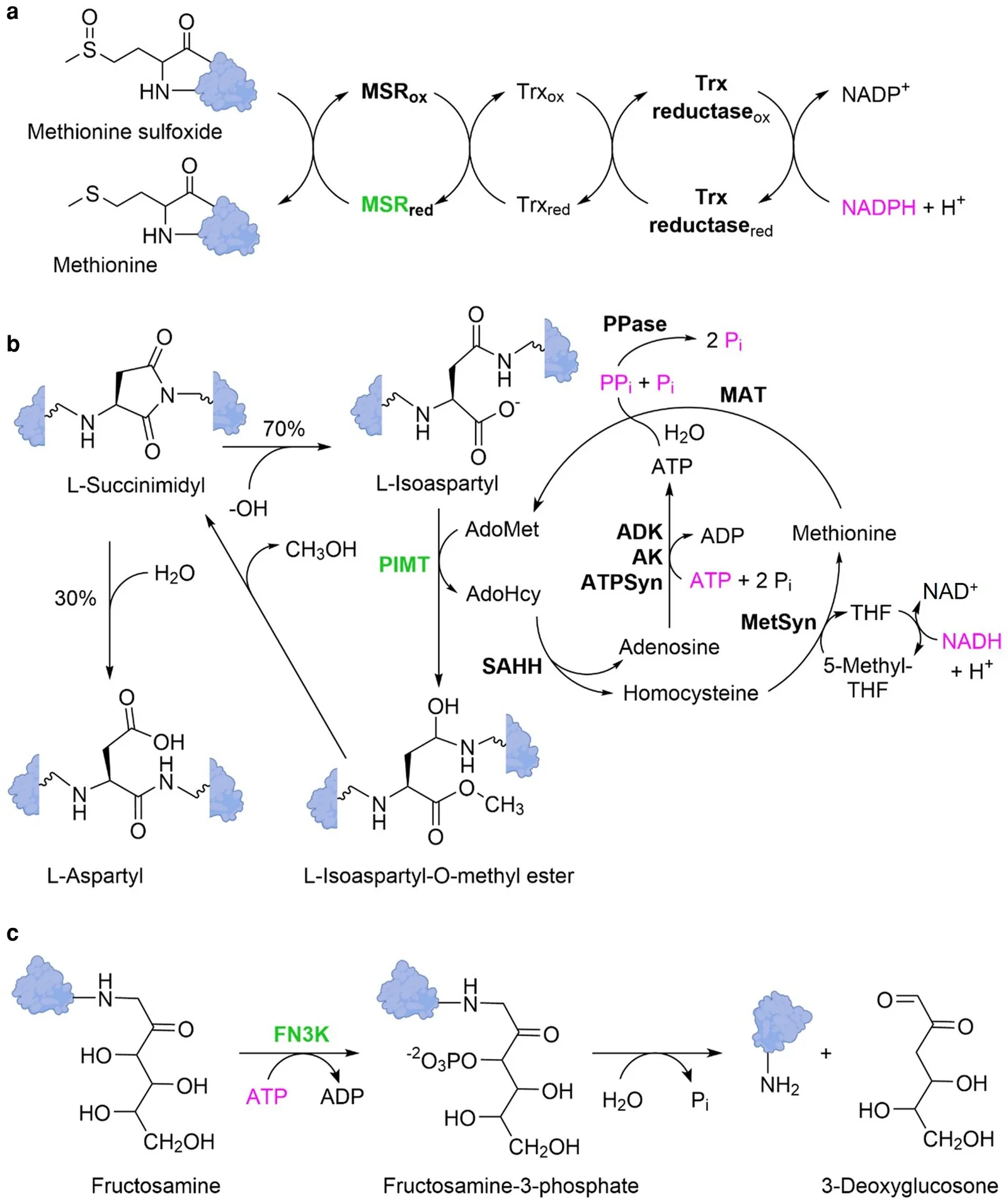
Protein repair and related costs. Many protein repair mechanisms require direct or indirect energy input (highlighted in pink). a) MSRs reduce free or protein-bound Met-O. The recovery of its catalytical cysteine residues involves the Trx or Grx systems and NADPH. b) L-Succinimidyl is repaired by PIMT. The conversion requires 6.5 ATP equivalents per 0.3 repaired L-aspartyl. Per fully repaired product, ∼22 ATPs are consumed. c) FN3K recovers proteins from sugar-based damage using one ATP per deglycation. PPase: pyrophosphatases; MAT: methionine adenosyltransferase; ADK: adenosine kinase; AK: adenylate kinase; ATPSyn: ATP synthase; AdoHcy: S-adenosylhomocysteine; SAHH: adenosylhomocysteinase; MetSyn: methionine synthase; THF: tetrahydrofolate.

^b^AdoMet is regenerated from S-adenosylhomocysteine, which is cleaved into adenosine and homocysteine. One 5-methyltetrahydrofolate is required for the conversion of homocysteine to methionine, and the regeneration of this cofactor consumes one NADH. Adenosine is converted into ATP (consuming one ATP equivalent) and finally linked to methionine to produce AdoMet. This last step consumes 3 ATP equivalents (Fig. 3).

^c^Direct repair costs here refer to the consumption of ATP, NADPH, etc. during catalysis. Indirect costs, such as the synthesis of the repair enzyme itself, are distributed across multiple catalytic cycles and are thus excluded from this relative comparison.

The reactive carbonyl compound methylglyoxal can be detoxified via the glyoxalase pathway, in which glutathione cooperates with glyoxalase I and II to convert it into D-lactate (Alam et al. 2024). Subsequently, D-lactate is oxidized to pyruvate and channeled into the TCA cycle. However, detoxifying methylglyoxal puts glyoxalase I at risk of damage. Indeed, several homologs of Arabidopsis glyoxalase I were found to exhibit CCR values roughly 100-fold lower than the median CCR of Arabidopsis enzymes (Hanson et al. 2021).

## Protein repair mechanisms and the cost benefit over resynthesis

Protein repair enzymes andmolecular chaperons help to prevent or reverse protein damage; thus, they have pivotal functions in maintaining proteome integrity to allow photosynthetic stability, growth and development, and stress tolerance. Some of them are conserved across kingdoms (eg MSR and Hsp70), and they provide cost benefits over protein degradation and de novo synthesis. Table 1 exemplifies repair enzymes and chaperons with direct and indirect (eg for cofactor repair) cost estimations. In the text below, we highlight as important examples the function, mechanism, and energy requirements of a few of them, namely MSR, protein-L-isoaspartyl methyltransferase (PIMT), and fructosamine-3-kinase (FN3K).

Oxidation of methionine residues can have diverse regulatory functions, eg to switch protein function on and off (Xiao et al. 2021), to prevent *O*-phosphorylation when located next to regulatory residues such as serine, tyrosine, and threonine (Rao et al. 2015), and to protect critical residues from damage (Levine et al. 1999). However, excess oxidation might inactivate proteins, and MSR helps to reduce Met-O in a stereospecific manner. For example, MSRs of type A convert Met-*S*-O, and type B specifically act on Met-*R*-O (Vieira Dos Santos et al. 2005; Laugier et al. 2010). The reduction involves catalytic cysteine residues in MSRs, which first form sulfenic acid and then an intramolecular disulfide (Fig. 3a). Although this reaction needs no energy input, the regeneration of MSRs does; it involves Trx or Grx under consumption of NADPH to restore its active state (Laugier et al. 2010). Depending on protein size and the number of methionine residues repaired, the cost benefit relative to degradation and resynthesis might be up to a thousand times (Table 1).

Isoaspartate formation is typically linked to damage because it introduces kinks into the protein backbone causing misfolding and inactivation (Ogé et al. 2008). Using the cofactor *S*-adenosyl methionine (AdoMet), PIMT can repair antioxidant enzymes such as SOD and CAT (Ghosh et al. 2020). During catalysis (Fig. 3b), L-isoaspartyl is methylated, and the subsequent spontaneous ester hydrolysis forms the metastable succinimide intermediate (Young et al. 2005). This reaction is only 30% effective because the L-succinimidyl hydrolyzes in a 70:30 ratio to L-isoaspartyl and aspartyl. Consequently, the energy requirement per fully repaired L-isoaspartyl residue is ∼22 ATPs equivalents, which is used for cofactor regeneration (Table 1; Fig. 3b).

Protein glycation can be an important feature in biotechnology and pharmaceutical industry to achieve protein stability and other properties. In cells though, this spontaneous chemical reaction is harmful and must be limited or repaired (Chaplin et al. 2019). FN3K and related enzymes have been identified in plants and other domains of life (Shrestha et al. 2020; Garg et al. 2025), in which they play important roles to reverse sugar-based protein damage (Fig. 3c). The reaction involves transfer of the γ-phosphate group from ATP to the fructosamine, and the resulting fructosamine 3-phosphate is instable decomposing to 3-deoxyglucosone, inorganic phosphate, and the repaired protein (amine) (Shrestha et al. 2020). 3-Deoxyglucosone is a dicarbonyl compound and very reactive; thus, it must be detoxified, for example, by the glyoxalase system, which consumes additional energy (Ghosh et al. 2016).

## Impact of environmental stress on protein turnover

### Protein turnover in changing environments

**Figure 4:**
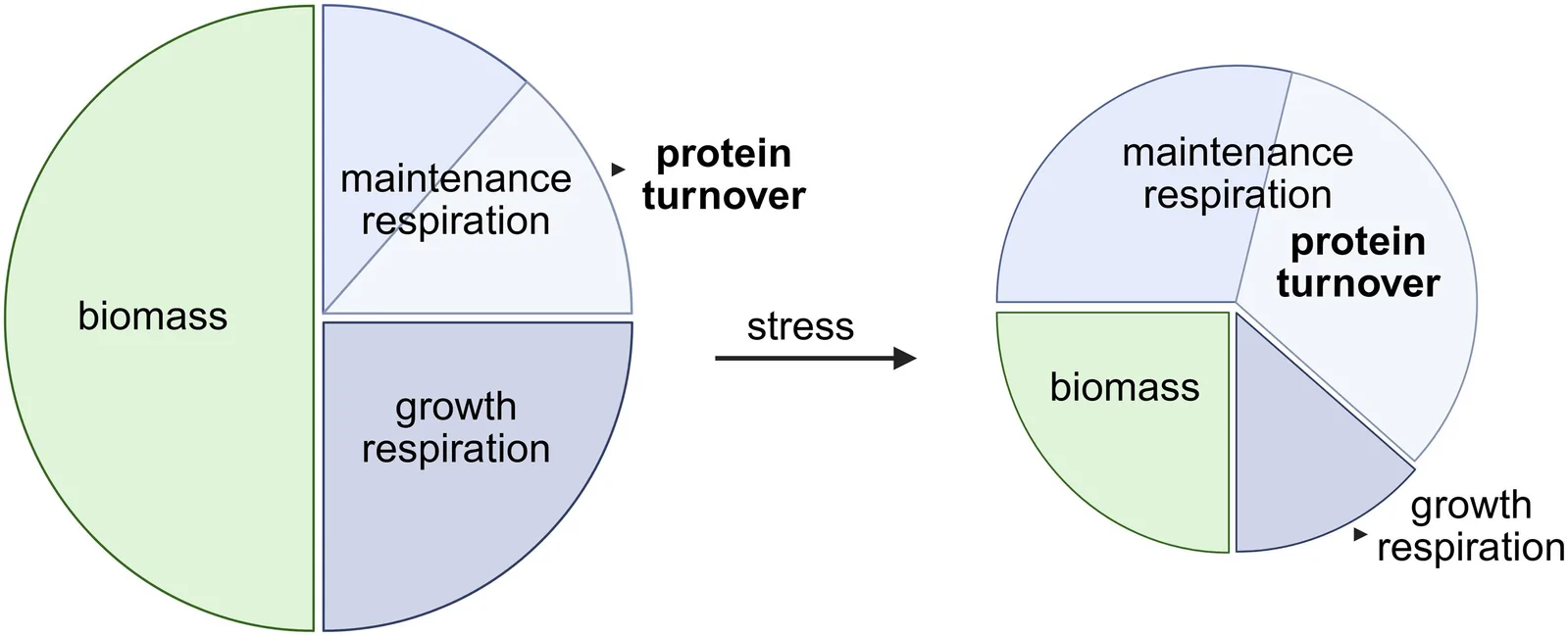
Costs of protein turnover under stress conditions. Physical and chemical stress can impact biomass production owing to increased maintenance respiration and reduced photosynthesis rate (the circle area is proportional to total fixed carbon). Slowed photosynthesis leads to less carbon uptake, which is depicted here by a smaller pie on the right side. Under mild or moderate biotic stress, plants might keep up or even temporarily increase their photosynthesis rate. The figure was created in BioRender (https://BioRender.com/bojpga9).

Protein turnover rates and related costs are influenced by growth conditions and environmental variability. Biotic and abiotic stress can increase protein turnover and respiration, thus reduce potential biomass production and consequently yield (Fig. 4) (Hachiya et al. 2007). For example, respiration rates upon infection were found to be increased by an average of about 26% across 115 studies, and elevated temperature causes rice yield loss perhaps related to increased respiratory rate (Li et al. 2021; Zhang et al. 2022). The increased protein turnover rate under environmental stress has 2 major causes. (i) Acclimation to environmental changes requires adjustment of the proteome, and protein degradation is important to regulate cellular function and promote survival. As a result, protein quantities, their localization, and their modifications are tuned, causing degradation of certain proteins and synthesis of others to meet the plant's changing needs (Fan et al. 2016; Jain and Schmidt 2024; Kusá et al. 2025). (ii) Changes in temperature and the redox environment can lead to more protein damage from both outside and inside the protein. For example, increased temperature favors faster enzyme reactions and changes the rate of metabolism (Arnoldi et al. 2025). This speeds damage from “inside,” eg “mechanism-based self-inactivation” (Bathe et al. 2021), and can increase respiration rates (Louwerse et al. 1990; Di Matteo et al. 2018; Rashid et al. 2020). If temperature increases further, protein denaturation and misfolding can happen. To maintain cellular function and to recover nitrogen from damaged proteins, degradation and de novo synthesis must follow—under cellular stress, this happens more often and with elevated costs.

### Protein turnover costs under abiotic stress

Plant cellular compartments that are most exposed to damage include the photosynthetic apparatus in chloroplasts. The protein D1 in PSII is the most labile part of the enzyme complex, and damaged D1, eg by tryptophan oxidation through photoinhibition (Kato et al. 2023; McKenzie and Puthiyaveetil 2025), is rapidly degraded and replaced by new molecules (Li et al. 2017). Accumulation of damaged D1, for example, in mutants that lack the involved proteases, not only impairs photosynthesis but also induces further damage. Therefore, quality control proteins (eg chaperons, proteases, and ROS scavengers) are upregulated (Dogra et al. 2019). D1 photooxidative damage is directly proportional to light intensity, and its half-life decreases with increasing light (Park et al. 1995). In fact, D1 has one of the highest turnover numbers of all plant proteins with a half-life of a few hours (Nelson et al. 2014; Li et al. 2017). Damage-induced inactivation of PSII proteins (like D1) is considered a protection mechanism to prevent damage on photosystem I (PSI) for which a repair system seems not to exist (Li et al. 2024). This is because electron flow from PSII to PSI is increased under high light causing ROS production and consequently irreversible damage in PSI (Yang et al. 2019). It is noteworthy that no direct repair mechanism at the polypeptide level is known for D1, instead that PSII repair involves degradation and de novo synthesis of D1 to be incorporated into the partially disassembled PSII complex (Chotewutmontri and Barkan 2020; Xu et al. 2021). In Table 2, we calculated the D1 turnover cost over 1 d using data from *Arabidopsis thaliana* grown under moderate and high-light conditions (Bielczynski et al. 2016; Graça et al. 2021; Li et al. 2022). We estimated that the ATP requirements under high light increase by ∼36%, matching the common assumption of “more light causes more damage” and consequently cost. We also found that the proportion of total ATP production devoted to D1 turnover remains constant at 0.5% (Yi et al. 2022). This is because plants adapt the photosynthetic apparatus to changing conditions, eg the data used for the calculation came from light-acclimated plants. The increased ATP production rate at higher light partially compensates for the costs of a faster D1 turnover, but other damage can co-occur in high-light conditions (eg by action of ROS), together reducing available energy for biomass and yield production. It can be expected that more extreme conditions, eg very low or excess light, shift the relative costs of D1 repair because insufficient energy production or photoinhibition, respectively, will become more prevalent.

**Table 2:** ATP cost estimation for D1 damage in *Arabidopsis thaliana*.

Calculation step	Low light	High light	Refs.
Chlorophyll content	175 µmol m^−2^	160 µmol m^−2^	Bielczynski et al. (2016)
Chlorophyll molecules	1.05·10^20^ m^−2^	0.96·10^20^ m^−2^	Calculated
Chlorophyll molecules per PSII RC	300	220	Graça et al. (2021); Bielczynski et al. (2016)
D1 molecules	3.51·10^17^ m^−2 a^	4.38·10^17^ m^−2 a^	Calculated
D1 half-life in light	4.38 h	1.61 h	Li et al. (2022)
D1 molecules replaced in 16 h	3.23·10^17^ m^−2^ h^−1^ (92%)	4.38·10^17^ m^−2^ h^−1^ (99%)	Calculated^b^
ATP cost per leaf area	7.2·10^20^ m^−2^ h^−1^	9.8·10^20^ m^−2^ h^−1^	Bathe et al. (2023); The UniProt Consortium (2018)
Repair cost increase	—	36%	Calculated
Light intensity	200 μmol photons·m^−2^·s^−1^	600 μmol photons·m^−2^·s^−1^	Li et al. (2022); Bielczynski et al. (2016)
Photons produced within 16 h	6.94·10^24^ m^−2^	20.81·10^24^ m^−2^	Calculated
ATP production within 16 h	8.67·10^23^ m^−2^	26.01·10^23^ m^−2^	Amthor (2010)
Relative ATP cost	0.083%	0.037%	Calculated

The calculations were made on the basis of data from light-acclimated plants grown in 16 h of moderate (100 to 200 μmol photons [photosynthetically active radiation {PAR}] m^−2^ s^−1^) and high (500 to 600 μmol photons [PAR] m^−2^ s^−1^) light. Numbers dependent on growth conditions, developmental stage, and species, though the calculated values indicate the relative ATP costs. RC: reaction center.

^a^Consistent with literature values (Bielczynski et al. 2016). PaxDB: 4.1·10^17^ m^−2^ in Arabidopsis leaves.

^b^ *k* = ln2/half-life in light; D1 molecules replaced in 16 h = D1 molecules × *e*^−k^  ^×^  ^16 h^.

Unless protein degradation has a particular cellular function (such as D1 degradation protects PSI), repair can generally be considered beneficial because it is more cost-effective than degradation and resynthesis. For example, the chaperon Hsp70 is a heat shock protein involved in unfolding preaggregated polypeptides (Aghaie and Tafreshi 2020) (Table 1). It consumes 5 ATPs to repair a protein substrate (Sharma et al. 2010); thus, the energy requirements are ∼1/700 of that for degradation and resynthesis. This example demonstrates that protein damage under abiotic stress is a major threat to the plant cell's functionality (Luciński and Adamiec 2023), leading to yield losses (Gietler et al. 2025). Conversely, repairing damaged proteins instead of rebuilding them conserves metabolic resources, which can promote biomass accumulation.

### Amino acid oxidation drives protein turnover under biotic stress

Biotic stress, such as pathogen infection, causes proteome changes, enabling plants to counteract the aggressor on the molecular level (Xie et al. 2025). The “oxidative burst” is an early response following pathogen recognition and releases ROS, often to the apoplast, to inhibit pathogen growth (Yang and Fernando 2021). From the apoplast, hydrogen peroxide diffuses across membranes to other tissues where it regulates defense reactions and mediates pathogen resistance (Bienert et al. 2007; Cao et al. 2024). Oxidation of proteins plays a crucial role, for example, that of cysteine amino acid residues. The cross talk between oxidized cysteine and *O*-phosphorylation as signaling mechanism is well established (Ma et al. 2024; Lu et al. 2026), and several global proteomics analyses have investigated cysteine oxidation patterns to identify defense-related targets (Wang et al. 2022b; Chen et al. 2023). Importantly, cysteine oxidation is reversible involving cellular redox systems, eg the Trx and Grx systems (Hou et al. 2024). The related costs are 1 NADPH per disulfide bond, which are associated with the recovery of Trx and Grx (Table 1; Fig. 3). These systems provide on–off switches for signaling and protect proteins from permanent oxidative damage. However, when the oxidative burst is very strong or lasts too long, irreversible damage occurs.

Although methionine is well known for its oxygen sensitivity, its redox chemistry during biotic stress has not yet been fully characterized. In contrast to cysteine, global proteomics of methionine oxidation upon infection are absent in the literature. This might be because Met-O measurements are technically challenging. Next to regulatory functions, oxidized methionine is among the most abundant protein modification markers under oxidative stress; thus, it can be considered a major cause of protein damage (Hoshi and Heinemann 2001; Bechtold et al. 2009). Both free and protein-bound Met-O can be reduced by MSRs (Table 1) (Rey and Tarrago 2018). Therefore, MSRs contribute to restore protein function and help to limit protein degradation and associated turnover costs. Under abiotic stress conditions, MSR overexpression supports tolerance to ROS, light, salt, and other stresses (Romero et al. 2004; Li et al. 2012; Ding et al. 2021, 2023; Wang et al. 2022a; Zhao et al. 2022a), and it might help to counteract pests as well (Rathinam et al. 2022, 2025). Salicylic acid, a key hormone in plant immune responses, triggers expression of MSR genes, and overexpression of a pepper MSR gene in tomato improved defense against *Phytophthora capsici* and *Phytophthora infestans* (Oh et al. 2010; Dai and Wang 2012). Additionally, MSR gene silencing increased disease symptoms upon *Botrytis cinerea* infection (Fu et al. 2023), and Arabidopsis MSR mutants and overexpression lines were more susceptible and resistant, respectively, against *Pseudomonas syringae* pv. *tomato* DC3000 (Roy and Nandi 2017). Loss of MSR function likely increases plant susceptibility to ROS by limiting the capacity to repair oxidized proteins, thereby delaying recovery; instead, damaged proteins accumulate and must be degraded (Romero et al. 2004; Bechtold et al. 2009). Well-characterized MSR targets are glutathione S-transferases which promote protection against (a)biotic stress (Tossounian et al. 2019). For example, glutathione transferase Phi 9 is oxidized at 4 methionine residues (Tossounian et al. 2019), and its repair by MSR costs 10 ATP equivalents (Table 1). The cost benefit over de novo protein synthesis (which is ∼1350 ATPs per molecule) is more than 100-fold. However, MSR can repair Met-O but not methionine sulfone (Min et al. 2023). Proteins containing fully oxidized methionine residues are damaged irreversibly, and the respiratory demand of their replacement can slow growth and development (Bechtold et al. 2004; Bathe et al. 2023).

## Potential of improving crops by engineering protein turnover and repair

### Why protein turnover and repair are good targets for crop improvement

Plants evolved under nutrient-limiting conditions, eg with a nitrogen and phosphorous deficit. The surplus of photoassimilates relative to other nutrients caused little or no need to evolve C-efficient (maintenance) respiration. Nutrient availability in crops has changed since the Green Revolution, as fertilization provides enough nitrogen, phosphorus, potassium, etc. Thus, modern crops can be C limited, eg during seedling development before canopies mature, during grain filling in high-yield varieties, in fruit crops with heavy fruit load, and under environmental stress. While growth respiration can unlikely be optimized because it supports biomass production and its efficiency is likely close to maximum in crops (Amthor 2025), maintenance respiration is a prime though underestimated and understudied target for plant engineering (Amthor et al. 2019; Garcia et al. 2023; Amthor 2025). The potential arises from needlessly high protein turnover. When combined with reduced “sink-limitations,” eg higher grain number, prolonging protein life and improved protein repair can be promising strategies to reduce respiration costs and to increase yield (Donald 1968; Burnett et al. 2018).

Turnover rates, abundance, and size of proteins correlate with metabolic costs, e.g., large proteins that are required in high amounts and quickly turn over are exceptionally expensive. The most costly proteins in Arabidopsis include Rubisco, Rubisco activase, thiazole synthase, and ATP synthase, which have ATP costs between 1.5 and 4.1 µmol g^−1^ FW d^−1^ (Bathe et al. 2023). Given that the daily ATP production is in the range of ∼500 to ∼7,000 µmol g^−1^ FW d^−1^ (Li et al. 2017; Cao et al. 2022), the above numbers for single proteins consume a substantial portion of the daily energy budget (see also Table 2). Replacing damage-prone amino acids in short-lived enzymes may increase their working lifetime and thus reduce respiration rates (Bathe et al. 2021). The yield gain has been estimated to be in the range of 2 to 4% for single or multiple proteins depending on degree of lifetime extension (Hanson et al. 2018; Bathe et al. 2023). By comparison, yield improvements of the major crops maize, rice, wheat, and soybean achieved by current breeding strategies are ∼1% per year (Ray et al. 2013; Andrade et al. 2026). Another target to decrease maintenance costs in nonharvestable crop organs is the reduction of protein size (Holland et al. 2019). A lower number of amino acids per protein minimizes the total ATP costs for degradation and de novo synthesis. Additionally, smaller enzymes need fewer macroelements (C, N, O, H, etc.), which reduces fertilizer requirements. Inspired by the concepts of “minimal cells” and “minimal genomes,” minimal proteins might be attractive for future plant applications (Hutchison et al. 2016; Moger-Reischer et al. 2023; Huang et al. 2025).

Overexpression of genes has been proven beneficial for plant performance under biotic and abiotic stress conditions (see Amino acid oxidation drives protein turnover under biotic stress), but it might have disadvantages, including: (i) increased cost for protein production and maintenance using resources that might otherwise be used for biomass production, and (ii) decoupling of expression from native regulatory mechanisms risking disturbance of tightly regulated pathways. Therefore, expression of improved enzymes under their native gene regulation is a more elegant approach. It allows for expressing repair enzymes with elevated catalytic efficiency when physiologically needed. For example, Met-O reduction is catalytically only about 1% as efficient as other reactions involving sulfenic acid intermediates such as peroxidases (Crane et al. 2000). Consequently, there might be room for improvement. Engineering plant MSRs and other repair enzymes could accelerate protein recovery upon damage and therefore improve plant performance.

### Synthetic biology to achieve the required changes in crops

Synthetic biology (SynBio) aims at engineering single biological parts, individual organs, or whole organisms. Tools such as genetic circuits, prime editing, synthetic transcription factors, and artificial biosensors have paved the way to (re)shape biology for customized functions across life kingdoms (Park et al. 2024; Simmons et al. 2024; Pierce et al. 2025; Chacko et al. 2026). They are expected to make major contributions in plant biology too. Through the establishment of precise genome editing (GE) tools, eg CRISPR/Cas, plant SynBio has made a significant step towards efficient and sustainable crop improvement.

A powerful SynBio approach to achieving protein changes is directed evolution (DE). It includes iterative hypermutation, translation, and selection that can be done in vitro*–*in vivo (classical DE) or entirely in vivo (continuous DE [CDE]) (Wang et al. 2024; Diercks et al. 2025). By using fast-propagating hosts and accelerated mutation rates, DE outperforms natural evolution in annual crops (eg maize) by up to 10^8^ per unit time (Clark et al. 2005; Rix et al. 2023). The combination of DE and GE (DE–GE) in plants has been considered valuable and reviewed multiple times (Gionfriddo et al. 2019; Rao et al. 2021; Kababji et al. 2024; Gayen et al. 2025). The pipeline would include engineering in vitro or in a microbe, followed by biochemical characterization and *in planta* testing, with bioinformatic and other artificial intelligence (AI)-based tools supporting at various steps (Mao et al. 2017; Wu et al. 2019; Qian and Shi 2024; Reed et al. 2024; Thomas et al. 2025).

Very recently, a geminivirus replicon–assisted *in planta* directed evolution (GRAPE) system allowed screening of variants in *Nicotiana benthamiana* via *Agrobacterium*-mediated transformation (Zhu et al. 2025). One round of evolution takes 8 d from building a mutant library to the final variant selection. The technique is a breakthrough in plant SynBio because it allows screening of target variants *in planta* on a reasonable timescale. The turnaround time lags behind microbial in vivo DE systems, however, which perform one evolution cycle per cell division. With mutation rates of 1 million times the genomic mutation rate, OrthoRep in yeast has proven to be exceptionally powerful (Rix et al. 2023). This and other microbial CDE systems have the advantage of easy access to knockout mutants and other genome edits required for selection of (plant) mutant variants. Recently, OrthoRep has been used to extend the working lifetime of Arabidopsis histidinol dehydrogenase (Oliveira-Filho et al. 2025). Furthermore, MutaT7 systems were applied to engineer Rubisco and arogenate dehydratase (Leong and Hanson 2023; McDonald et al. 2025). Although none of the improved protein variants generated by CDE have been tested *in planta* yet, such approaches provide novel strategies to improve crops.

While directed evolution offers great potential for engineering crops, applying these strategies to mitochondrial and chloroplast proteins can be challenging. For example, posttranscriptional C→U RNA editing in plant mitochondria and chloroplasts uncouples the genomic DNA information from the final protein sequence (Covello and Gray 1989; Hoch et al. 1991). Consequently, organellar base substitutions can potentially reverse engineered mutations to restore wild-type amino acids, introduce unpredicted mutations, or disrupt the precise binding motifs required by nuclear-encoded pentatricopeptide repeat (PPR) proteins (Small and Peeters 2000). However, RNA editing holds potential to be exploited and redesigned for custom knockdown or switch-on of mitochondrial and chloroplast transcripts using synthetic PPR proteins (reviewed by Small et al. 2020).

Recently, diverse studies reported engineered degradation of proteins by inducible or target-specific systems. Using phage-assisted continuous evolution (PACE), Mercer and coworkers evolved small molecule–triggered degrons that recruit proteins for degradation by the ubiquitin-proteasome system (Mercer et al. 2024). This enabled the selective degradation of proteins in response to a thalidomide derivative. In another study, glucocorticoid-activated E3 catalytic activity (E3-DART) was engineered in plants based on protein interactions of a *Salmonella* protease and the human protein kinase N1 (Huang and Rojas-Pierce 2024). Furthermore, Luo and colleagues demonstrated increased tiller production and enhanced pathogen resistance in rice utilizing a degradation system that targets proteins prone to condensation (Luo et al. 2025). Notably, peptides that derive from mitochondrial degradation could act as retrograde signals to both the nucleus and mitochondria (Møller and Sweetlove 2010). Manipulation of this degradation may have off-target effects and must be studied carefully. However, in engineered crops, such efforts could allow us to switch on or off protein degradation at specific developmental stages, activate or deactivate pathways on demand, fine-tune flower development and seed setting, induce stress tolerance, or control fertility/sterility.

AI-driven approaches, automated systems, and technology to scale up the collection of laboratory data as well as extended genetic codes for novel nucleotides and amino acids will further drive advancement in (plant) bioengineering (see Outstanding questions) (Sawada et al. 2025; Shen et al. 2025; Tran et al. 2026). What we need now is a reduction in the regulatory barriers of genetically modified crops that will allow us to bring plant innovations into practice.

## Concluding remarks

Protein turnover represents a major sink of respiratory C in plants. The continuous degradation and de novo synthesis of damaged proteins is energetically costly, and the C required to fuel protein replacement cannot be allocated to yield production. Consequently, protein turnover represents a limitation on plant productivity. This yield penalty becomes more severe under environmental stress conditions, which increases protein damage and thereby accelerates turnover rates. There is growing evidence that protein turnover costs in plants have not been fully optimized during evolution. However, plants have a few molecular mechanisms that prevent or repair protein damage, thereby reducing respiratory costs. Importantly, these mechanisms seem to be exceptions rather than the rule because most proteins are replaced upon damage, leading to unavoidable C losses. In the context of modern agriculture, this issue has particular importance. Many contemporary crop systems are likely C limited, yet current breeding strategies are struggling to deliver yield gains that will meet future global demand. This mismatch highlights the need to explore alternative targets for crop improvement beyond traditional traits. Protein damage and repair pathways in plants remain relatively understudied, especially for applications using SynBio. They represent promising and largely untapped opportunities for engineering crops with reduced respiratory losses, improved C-use efficiency, and ultimately higher and more stable yields.

Advances Box

- Protein damage is critical in plants because it consumes substantial respiratory energy and can restrict crop yield.
  • Advances in mass spectrometry–based proteomics now enable robust analysis of protein damage and turnover at the molecular level.
- Plants have evolved molecular mechanisms to prevent and repair protein damage, providing an energy benefit over resynthesis.
- Abiotic and biotic stresses increase protein damage, imposing respiratory costs that exceed the energy needed for regular protein degradation and de novo synthesis.
- Synthetic biology offers new avenues for engineering plants and can provide innovative strategies for improving crops, eg to cut respiratory costs of protein turnover.

Outstanding questions box

- How do accidental and regulatory protein modifications integrate to shape cellular and whole-plant physiology?
- How do stress conditions (eg extreme weather and pests) modulate protein turnover, and how can these insights be applied to engineer resilient future crops?
- Which structural features can protect essential, but vulnerable, amino acid residues from damage?
- What protein sites for engineering can be predicted by leveraging scale-up of data collections (eg high-throughput continuous directed evolution) to train machine learning models.
- To what extent can extending enzyme half-life and fine-tuning protein degradation enhance overall crop yield?
- In what ways can gene editing frameworks be optimized to accelerate innovation and the implementation of SynBio in agriculture?

## Data Availability

There were no new data generated for this article.
